# Development and evaluation of a restaurant virtual reality training system for enhancing awareness and priority-setting skills

**DOI:** 10.1038/s41598-025-00194-0

**Published:** 2025-05-28

**Authors:** Mai Otsuki, Takashi Okuma

**Affiliations:** National Institute of Advanced Industrial Science and Technology, Kashiwa, Japan

**Keywords:** Virtual reality, Training, Restaurant, Awareness, Priority-setting, Electrical and electronic engineering, Mechanical engineering

## Abstract

Characterizing trainees’ cognitive and decision-making processes presents a challenge for trainers, hindering effective on-the-job training (OJT) in the restaurant industry. Thus, objectively evaluating training effectiveness proves to be difficult. To support aspects that are difficult to address through the current OJT, we developed a job-training system based on virtual reality (VR). The system includes both a training mode and a scoring mode. In training mode, trainees wore a head-mounted display (HMD) and held the controller in both hands to perform operations in a virtual restaurant. In scoring mode, instructors could view replays of the trainees’ operations on a PC screen and provide comments. This system targets two elements that pose challenges in real-world scenarios: awareness and priority-setting. Given the importance of evaluating the training effectiveness and user usability of the training system, we designed and conducted an evaluation experiment with 50 novices and 20 experts. Although scores before and after training exhibited no significant differences in the tests using videos recorded in actual settings, both novices and experts highly praised the system’s utility.

## Introduction

In Japan, labor shortages across various industries are escalating, exacerbated by a shrinking working population. A survey by the Japan Research Institute, Ltd. found that the effects of the COVID-19 pandemic increased the severity of the situation, especially in service industries involving face-to-face interactions, such as the hospitality and food sectors^1^. Similar problems have been reported in Europe and the United States, with the destabilization of industries due to lockdowns and the aging and population decline cited as causes^2,3^. Our focus is on studying technologies aimed at enhancing operational efficiency to mitigate labor shortage^4^.

As do many others, the restaurant industry involves simultaneous operations, such as manufacturing, transportation, customer service, consumption, and collection. Consequently, our empirical research is centered around identifying support technologies that are applicable across a broad spectrum of service industries, particularly focusing on food and beverage service sites.

Customer service training in the restaurant industry generally takes the form of on-the-job training (OJT), wherein an instructor accompanies the trainee and guides them during actual customer interactions. Although coaching someone on serving a single table through observation may appear straightforward, challenges arise when dealing with multiple tables, such as what one should be aware of (awareness) and how the worker should proceed based on what has been noticed (priority-setting) for multiple tables. It is difficult for instructors to objectively determine the progress of trainees in cognition and judgment needed to answer these questions; thus, OJT is extremely demanding. In addition, an objective evaluation of training effectiveness requires quantitative metric measurements that do not depend on the instructor; however, performing measurements while serving customers at an actual restaurant is difficult. Furthermore, under the current conditions of labor shortages, experienced staff face challenges in consistently attending to trainees and spending time instructing them.

To support aspects that are difficult to address through the current OJT, we developed a job-training system based on virtual reality (VR). VR training has the following advantages: it reduces the cost of establishing training facilities, preparing training materials, and hiring full-time instructors; it allows for three-dimensional, interactive explanations of skills and processes; it facilitates safe training for crisis response, makes repetitive training easier, and enables objective performance evaluation and immediate feedback of results^5–10^. In a restaurant case, Flintham et al.^11^ developed a VR game for kitchen staff to learn about hygiene.

Major training topics in the hospitality industry include one’s attitude and way of speaking while serving customers, as well as handling customer complaints. For example, Iida et al.^12^ developed a VR system that can train restaurant floor staff how to use the difficult system of the Japanese honorific language. Fujita et al.^13^developed a system for training restaurant floor staff in a variety of scenarios, including handling complaints, a task that is difficult to train. Commercial customer service training systems also exist^14^.

Restaurant customer service staff must handle various tasks that occur simultaneously at multiple locations within the facility. To provide service without making customers wait, two skills are necessary: to be “aware” of the customers and situations in the restaurant, and to appropriately “prioritize” based on what is noticed.

This study proposes that by improving these awareness and prioritization skills using a VR system, the cognitive load on trainees when performing routine tasks can be reduced, freeing up the trainees to better handle complaints and irregularities. Past research on prioritization skills has examined triage following a large-scale accident^15^, but there have been no studies of the restaurant industry.

The effectiveness of commercial VR training systems in the restaurant industry has been evaluated, demonstrating results such as “an n%” reduction in training time and a reduction in instructor costs; however, the evaluation process is unclear^16^. Indeed, there are many cases, even in academic research, where evaluations are limited to qualitative aspects using interviews and questionnaires^11,13^.

The rationale for this study is based on the necessity to improve operational efficiency and address labor shortages in the restaurant industry through enhanced training methods. The hypothesis is that a VR-based training system can effectively improve awareness and priority-setting skills that are difficult to address through current OJT among trainees.

**Fig. 1:**
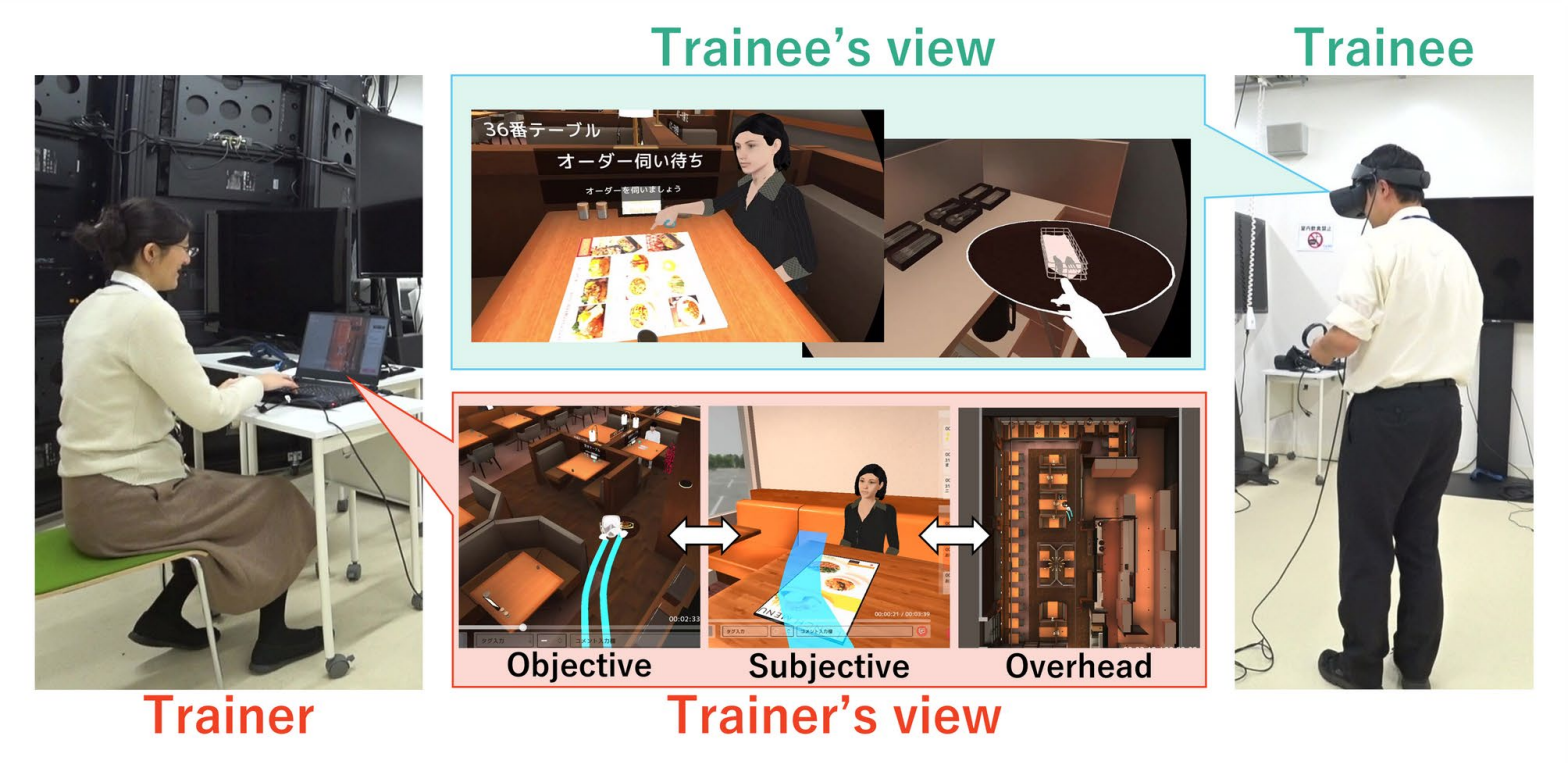
System overview.

This study contributes by focusing on the development of a VR-based training system that targets these skills (Fig. 1) and provides detailed methodologies for evaluating its effectiveness and usability. The system includes both a training mode and a scoring mode. In training mode, trainees wore a head-mounted display (HMD; Meta, Meta Quest 2) and held the controller in both hands to perform operations in a virtual restaurant. In scoring mode, instructors could view replays of the trainees’ operations on a PC screen and provide comments.

As mentioned, many restaurant VR training systems have been developed for both research and commercial use, but none focuses on awareness and prioritization skills. Moreover, cases that provide a detailed description of how the effectiveness and usability of the training system were evaluated are few. To improve the system, it is important to investigate the implementation results and the various factors related to those results, from both subjective and objective perspectives. Few studies explain the thinking upon which they were designed and implemented. This study addresses these gaps by offering a comprehensive analysis of the design and implementation, thereby contributing to the discussion of generalizability and interpretability of VR training systems in the restaurant industry and, by extension, to the broader field of customer service training.

## Results

The effectiveness, usefulness, and usability of our proposed restaurant VR training system were evaluated using training tasks in the Proof of Concept (PoC) system with the cooperation of participants who had a high degree of experience in restaurant customer service (experts) and novices. Experts participated for only one day and evaluated the system from an instructor’s perspective. Novices participated for five days and trained using the system. Awareness tests were conducted for novices on the first and final day (Day 5) to measure how participants changed before and after training. In addition, we collected the opinions of participants and compared how experts and novices used the system in the scoring mode for further system improvement.

A total of 20 experts (3 in their 20 s, 6 in their 30 s, 6 in their 40 s, and 5 in their 50 s) with teaching experience in the food and beverage services industry and 53 novices with no relevant experience were allowed to participate after providing informed consent. Of the 53 novices, seven were excluded owing to procedural errors and five withdrew owing to VR sickness; thus, 41 participated in the experiment (9 in their 20 s, 11 in their 30 s, 12 in their 40 s, and 9 in their 50 s).

### Subjective evaluation

**Fig. 2:**
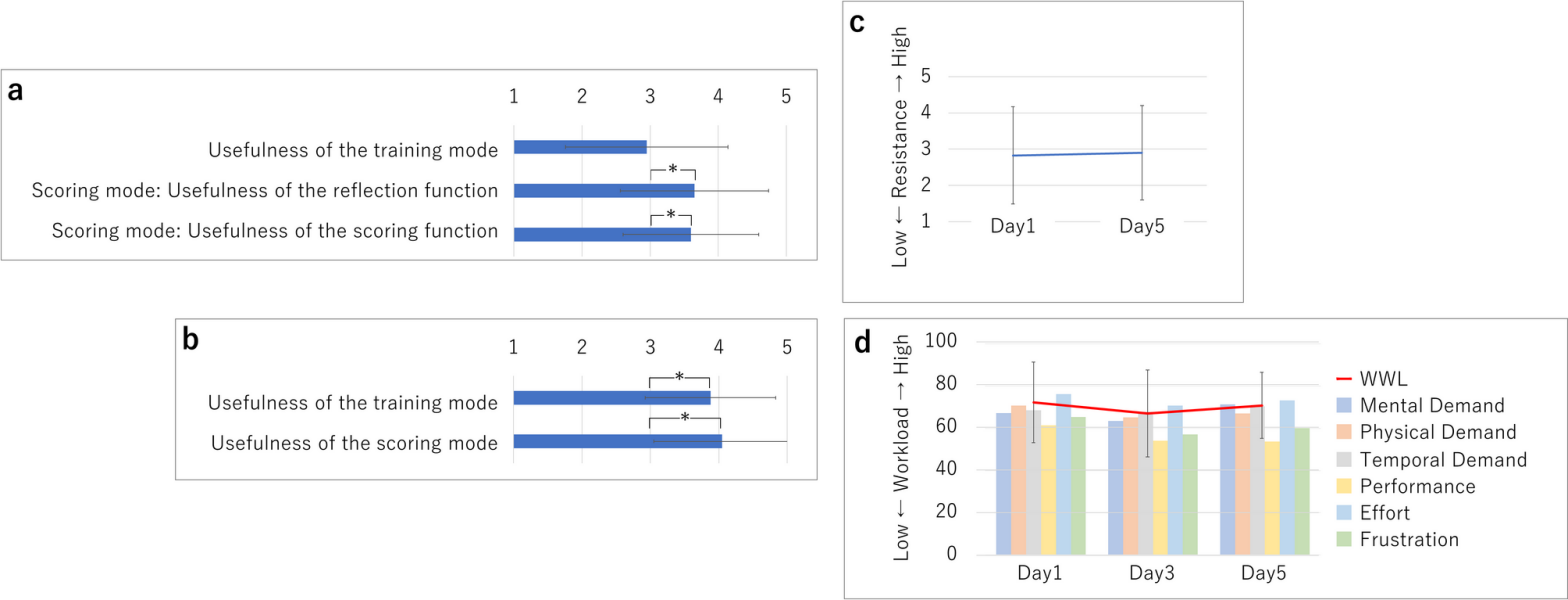
(**a**) Expert: Subjective evaluation results, (**b**) Novice: Subjective evaluation results, (**c**) Novice: Resistance to customer service, (**d**) Novice: NASA-TLX transition.

From the results of the subjective evaluations by experts (Fig. 2 (a)), one-sample t-test shows that the usefulness of the training mode did not differ significantly from the expected value (3) (t (19) = 0.19, *p* = 0.85, Cohen’s *d* = 0.04), but evaluations of the usefulness of the reflection function in the scoring mode and of the scoring function were significantly high (t (19) = −2.67, *p* = 0.015, Cohen’s *d* = 0.60, and t (19) = −2.70, *p* = 0.014, Cohen’s *d*= 0.60, respectively). Meanwhile, the average system usability scale (SUS)^17^result was 42.16 (SD: ± 17.88); a low value considering that Lewis et al. recommend 68 or higher^18^.

Figure 2 (b) shows the results of the subjective evaluation by novices on the usefulness of the training mode and the usefulness of the reflection function in the scoring mode. Both were significantly higher than the expected value (3) (one-sample t-test, t(40)=−5.90, *p* < 0.001, Cohen’s *d* = 0.9, and t(40)= −6.72, *p* < 0.001, Cohen’s *d* = 1.1, respectively).

There was no significant difference between the first and last day in feelings of resistance to customer service based on the Wilcoxon signed rank test (Fig. 2 (c)), between the first day and the last day, Z = 0.3, *p* = 0.739, effect size *r* = 0.07.

Positive results were obtained on all items regarding the iGroup Presence Questionnaire (IPQ)^19^with general immersion rated as “excellent” with a score of 5.4; remaining spatial presence, involvement, and experienced realism were rated “very good” with scores of 5.02, 4.78, and 3.88, respectively^20^.

The SUS of the novices was low both on the first day, with an average of 43.35 (SD: ±16.95), and on the last day, with an average of 54.39 (SD: ±15.74); however, it had significantly improved by the last day relative to the first day (Wilcoxon signed rank test, Z = 0.6, *p* < 0.01, effect size *r* = 0.74).

Figure 2(d) shows the results of NASA-TLX (Task load index)^21^ (39 participants for analysis, 2 with missing data), indicating the cognitive load in training mode. A one-factor analysis of variance performed on the weighted work load (WWL; total score) using the schedule as a factor found no significant differences between Days 1, 3, and 5 (F(2, 114) = 0.82, *p* = 0.44, $\:{\eta\:}^{2}$=0.014). Next, although the results of a 6 × 3 two-way analysis of variance performed using each subscale and schedule as factors showed no significant differences for the schedule factor (F(2, 76)=1.49, *p* = 0.076, partial $\:{\eta\:}^{2}$=0.04), significant differences were found between the subscales (F(5, 190)=7.48, *p*<0.01, partial $\:{\eta\:}^{2}$=0.16). The results of multiple comparisons (Bonferroni) showed that intellectual/perceptual demands, physical demands, time pressure, and effort were all significantly higher than work performance and frustration (all *p*<0.05). No interactions were observed (F(10, 380)=1.16, *p* = 0.96, partial $\:{\eta\:}^{2}$=0.03).

### Result of awareness test and number of points trainee became aware of during training mode experience

**Fig. 3:**
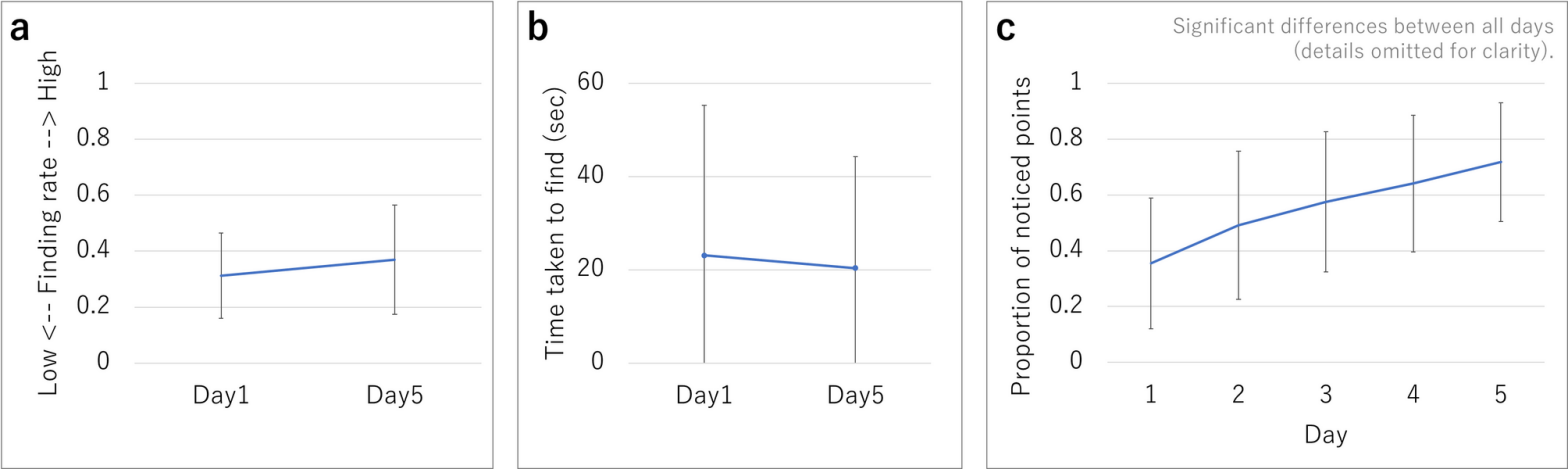
(**a**) Finding rate, (**b**) Time taken to find, (**c**) Novice: Proportion of noticed points during the training experience period.

In the 34 awareness tests in which participants’ voices were recorded without problems, Wilcoxon signed-rank test shows no significant improvement from training was observed in finding rate (z = 1.16, *p* = 0.246, Fig. 3 (a)) or time to find (z = 0.22, *p* = 0.8259, Fig. 3 (b)). The finding rate is defined as the proportion of predefined customer service key points noticed in the video.

In contrast, a one-way analysis of variance for the ratio of points trainees became aware of during the training mode experience (Fig. 3 (c)) with the number of days (it represents the sequential experimental days from the first to the fifth day of participation) as a factor found that it significantly increased each day (F(4,160) = 76.54, *p* < 0.01, partial $\:{\eta\:}^{2}$ =0.66).

### How experts and novices use scoring mode

**Fig. 4:**
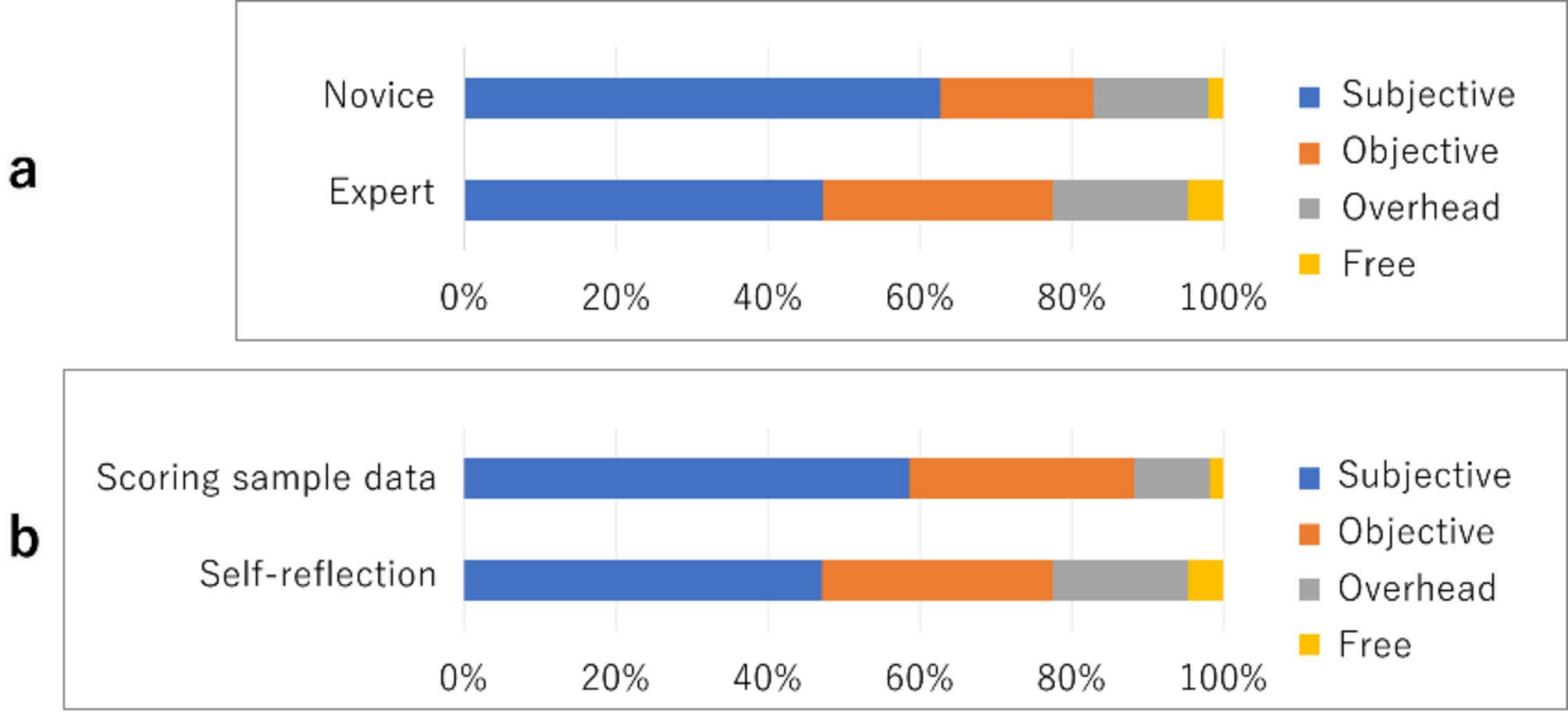
(**a**) Perspectives used during self-reflection. (**b**) Expert: Perspectives used during scoring sample data (= evaluation for others) and self-reflection.

Significant differences were observed in the results of a chi-square test performed on the percentage of time experts and novices spent using the four different viewpoints (subjective, objective, overhead, and free) in the scoring mode (Fig. 4 (a)) (*χ*^2^(3) = 52.101, *p* < 0.01, Cramer’s V = 0.165). Residual analysis results showed that novices used the subjective viewpoint for a significantly longer period than did experts and that experts used the objective viewpoint and free viewpoint for a significantly longer duration than did the novices.

Similarly, a chi-square test revealed significant differences in the percentage of time experts used each viewpoint for reflection and scoring (Fig. 4 (b)) (χ^2^(3) *=* 48.631, *p* < 0.01, Cramer’s V = 0.155). The results of the residual analysis showed that participants used the overhead and free viewpoints for significantly longer periods during reflection than during scoring. Furthermore, the subjective viewpoint was used for a significantly longer time during scoring than during reflection.

The average number of viewpoint switches during reflection was significantly higher for novices at 4.5 times, compared with 2.4 times for experts (Welch’s t-test, t(31) = 3.00, *p* < 0.01, Cohen’s *d* = 0.85). In addition, the average number of viewpoint switches by experts during reflection was 4.5, significantly higher than 1.9 during scoring (paired t-test, t(16) = 5.27, *p* < 0.01, Cohen’s *d* = 1.58).

The average number of comments entered in scoring mode was six during expert reflections and eight during scoring. In addition, reflections by novices averaged from eight to nine items/time, with a one-factor analysis of variance using day as a factor showing no significant change over the 5-day period (F(4, 160) = 0.11, *p* = 0.98).

The average time required for reflection was 1005 s for experts and 918 s for novices, with no significant difference observed (Welch’s t-test, t(22) = 1.38, *p* = 0.18. Cohen’s *d* = 0.48). Additionally, the time experts took for both reflection and scoring averaged 1000 s, with no significant difference observed (paired t-test, t(16) = 0.31, *p* = 0.76, Cohen’s *d* = 0.09).

### VR sickness

Among the experts, two experienced interruptions due to VR sickness. Among the novices, six experienced interruptions due to VR sickness. For five individuals, the interruption occurred only during the first trial, and four of these individuals could not resume on that day. The timing of the interruptions varied between individuals, with some occurring after approximately 2 min and others after approximately 9 min. No VR sickness incidents were reported after the second trial for them. For the remaining novice, interruptions occurred during both the first and second trials; however, both times the individual resumed after a 2-minute break.

## Discussion

In the subjective evaluation, only the novices indicated the usefulness of the training mode, while in the free description, both experts and novices positively commented on how they were able to learn the procedures before going out into the field, learn the lines of action and movements in the restaurant, and grasp the flow of customer service through the experience. Furthermore, IPQ results could be evaluated to determine whether the experiment achieved restaurant staff training in a VR environment that was highly immersive and realistic for novices.

**Fig. 5:**
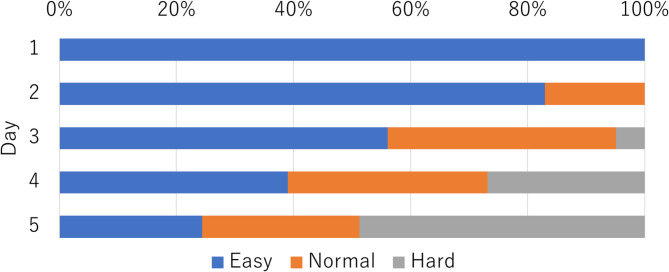
Proportion changes in difficulty over 5 days.

Regarding the training effectiveness, descriptions of “points trainees became aware of during the training mode experience” increased with the number of training days. Additionally, considering that the level of difficulty gradually increased based on the overall scores of participants, which resulted in half of the participants transitioning to “hard” on the last day (Fig. 5), it is possible that their skills improved even in such a short period, even though improvements in awareness test results were not observed. VR training makes it possible to grasp the situation inside the restaurant as a result of one’s work; however, it is possible that observing restaurant operations on video made it difficult to comprehend the context and thus to understand the situation, and that targets indicated on a screen—that can only appear small because the video was shot from a fixed location—were overlooked. Because the range of quantification targets for skills based on awareness tests designed to quantify the efficacy of the training in this study was set to a higher level of difficulty than skills that can be learned in short-term training, there is a possibility that the resolution to detect efficacy was insufficient. In addition, regarding the fact that resistance to customer service by novices did not decrease, participants commented about the differences with reality, such as concerns about lack of training in handling complaints, and the absence of other employees. In the hospitality industry, Fujita et al. proposed the VR system to train individuals how to respond to customer complaints^13^, thus it may be feasible to collaborate with such a system.

It was inferred from the SUS results and comments by participants that mental demand was high on the NASA-TLX analysis because it was necessary to constantly focus on the restaurant and the situations of its customers to set priorities and it may have been affected by the difficulty of operating the VR system. Additionally, high physical demands were likely because of VR sickness, while time pressure and effort reflected that the focus was on not making customers wait. Based on the NASA-TLX results, issues were identified in that there were some loads beyond the workload required for training, such as the difficulty of the VR operation and VR sickness. Elements that tend to cause motion sickness, such as floating objects or occlusion, were not present in the environment. We have also implemented methods to reduce VR sickness, such as the approach proposed by Fernandes et al.^22^, which involves narrowing the field of view during movement, reducing contrast during controller-based viewpoint rotation, and gradually increasing movement speed when starting to move with the controller. We enabled users to turn features on and off according to their preferences. However, in this experiment, it was necessary to standardize experimental conditions, and we considered that limiting certain visual information could potentially affect the training outcomes under investigation. Additionally, to accurately evaluate training effectiveness, the experiment was conducted under conditions that simulated real-world visual information as closely as possible. Consequently, we did not employ these methods for this particular experiment. Evaluating the effects of visual information limitations as methods for VR sickness on training outcomes is an area that requires further research.

Regarding the VR operation, VR sickness was almost non-existent during the second experience, and SUS scores significantly improved on the last day as compared to the first, suggesting that it is possible to master the system by getting used to operating it.

The above results indicate that although participants were able to learn the work procedures, understand the points to be aware of, and felt that the system was useful, the 5-day VR training did not lead to skill improvement or anxiety relief at levels detectable with current evaluation methods. In the future, when incorporating the proposed system into the educational curriculum, it is necessary to consider how often and for how many hours will be required for the training experience.

Regarding the scoring mode, positive evaluations were obtained from experts; these can reflect on experiences, provide feedback through comments, and give guidance afterward, particularly, given that restaurants are hectic places where appropriate decisions cannot always be made. Even novices evaluated the self-reflection function positively as it allowed them to replay one’s own actions from various viewpoints. Comments were also received about how quantifying achievement and failure makes it clear where improvements can be made, which leads to motivation for the next time. The aforementioned observations reiterate the importance of this study in overcoming the challenges of traditional in-person training, where the hectic nature of on-the-job situations and insufficient manpower makes it difficult to evaluate trainees and provide sufficient guidance, as well as the fact that the trainees can reflect and train themselves based on quantitative evaluations.

Regarding the function of observing by switching between the four subjective/objective/overhead/free viewpoints, this function was actively used as intended. For example, novices used the subjective viewpoint while experts experienced in teaching in the field tried to objectively retrospect on their own experiences, using an overhead or free viewpoint, and observe unknown-trainee’s experiences from a subjective viewpoint for long periods during scoring.

Several comments were given to trainees by experts in scoring mode. In “hosting,” about insufficient observation or delayed reception at the entrance, and prioritization of other tasks (e.g., receiving customers at the entrance before replacing the ice water at the table), delays in “taking orders,” and comments on the priority level of tasks. In addition, it was confirmed that comments in line with the training content were appropriately entered, including many comments about behavior, such as “being too close to customers;” comments about timing, such as whether the ice water replacement was done too early or too late, and comments about efficiency (e.g., if you’re going to clear empty dishes, bring out meals from the dish-up counter).

Because the actions of all the trainees and events can be recorded in VR, in the future it will be possible to obtain data to automatically prioritize for automatically determining the priority of simultaneous tasks. Future studies will gather scoring data from experts and implement an automatic evaluation function.

No significant differences were observed between reflection/scoring by experts and reflection by novices on the time required for scores. On average, for all participants, the 10-min (600-s) VR experiences was scored in 937 s (SD = 209 s), which is 1.6 times longer than the actual duration. Efficient scoring using the seek bar and cueing function from the list of restaurant events was initially expected; however, the experimental situation apparently made participants overtly conscious of the need to “look very carefully.” The results could be different if participants are instructed to score in the shortest possible time during the experiment, assuming they are using the system in the real restaurant training curriculum.

## Conclusion

This study proposed a VR training system that trains the restaurant service industry in the “awareness” and “prioritization” of events that currently happen at multiple locations throughout a restaurant. The system satisfies the following requirements:

(i) It must provide awareness and prioritization training.
(ii) It must objectively and quantitatively evaluate trainee responses.
(iii) It must enable instructors to qualitatively assess in real-time and non-real-time.

This paper describes the design and implementation of the system, as well as the design and implementation of experiments for the evaluation of system use.

The effectiveness, usefulness, and usability of our proposed restaurant VR training system were evaluated using training tasks in the PoC system with the participants cooperating with experts and novices. Although scores before and after training showed no significant differences in the tests using videos recorded in actual settings, the number of points trainees became aware of during the training mode experience significantly increased each day, and both novices and experts highly praised the system’s utility.

The contribution of this study lies in the development of a VR-based training system designed to enhance awareness and priority-setting skills in the restaurant industry, and in providing a detailed methodology for evaluating its effectiveness and usability.

Future work will expand the customer model in training mode. Specifically, we intend to make it possible to express the behavior of group customers, including how fast they make menu decisions and eat, and acceptable waiting times for each customer, based on the results of measuring customer behavior at actual restaurants^23^. Another challenge is to conduct training in more realistic situations, for example, with functions enabling multiple trainees to simultaneously participate in training and collaboration with other employees.

In this study, the only scoring criterion was whether the customer was kept waiting. Conversely, in real-world settings, there is an appropriate timing for restaurant tasks, such as intermediate bussing, that depends on the progress of the customer’s meal. Evaluation that considers such timing is the next challenge that we will take up for scoring mode. Furthermore, in terms of quantifying awareness skills, we are considering adding a function that uses an HMD to measure the trainee’s gaze and assess the proportion of attention paid to designated areas (entrances, tables, etc.), and evaluate whether trainees are observing the situation while efficiently moving around the restaurant.

We designed this system to support s to support aspects that are difficult to address through the current OJT. Another challenge is to evaluate the effects of installing the system in actual restaurants and introducing it into new employee training curricula with regular training. Future developments include the possibility of extending this system to other companies in the same industry and applying it to industries other than the restaurant industry, such as the childcare and healthcare industries.

## Methods

### Design of a restaurant VR training system

As discussed in Introduction, the VR training system proposed in this study must satisfy the following three requirements.

(i) It must provide awareness and prioritization training.
(ii) It must objectively and quantitatively evaluate trainee responses.
(iii) It must enable instructors to qualitatively assess in real-time and non-real-time.

First, regarding requirement (i), we set the basic structure of the training scenario for trainees to respond sequentially to customers who enter the VR restaurant at different times. By showing trainees the status of each customer and table as they change over time (e.g., thinking about their orders, eating), the system enables training in “awareness” of these changes and “prioritization” of the tasks that need to be addressed. In addition, the level of difficulty of the system could be set according to the proficiency level of the trainee.

Next, regarding requirement (ii), in terms of “always being aware of the customer situation, prioritizing it, and providing service without making the customer wait longer than necessary,” we adopted evaluation metrics based on the time elapsed from the change in customer situation or table state to the completion of a response, as well as the importance of that event. Because events requiring a response occur in parallel at multiple locations across the restaurant, we designed the system so that the total value of the evaluation index naturally rose when events were handled with appropriate “attention” and “prioritization.”

However, simply presenting quantitative evaluation results to trainees is not sufficient to help them understand how to improve, and a function that allows instructors to check training progress and provide qualitative evaluations and advice is necessary. Therefore, to fulfill requirement (iii), we provided a function that allowed instructors to observe the training in real-time, a function for recording the training process to review it later, and a function for giving evaluations and advice. Functions for controlling viewpoints and time were also provided to enable instructors to efficiently observe the training and recordings. Specifically, regarding the viewpoints, we provided a function that switched between four independent viewpoints: an objective viewpoint that simulated OJT instruction, a subjective viewpoint that allowed observation from the perspective of a trainee, an overhead viewpoint that allowed observation of the entire restaurant, and a viewpoint that could be moved to any position using the arrow keys. In addition, regarding the time, we provided a seek bar to observe any time during the recorded training, a list of events requiring responses that occurred during the training, and a function that allowed instructors to select an event to check the situation at that time.

### Implementation

Figure 1 shows an overview of the proof of concept (PoC) system, which was implemented based on the design in the previous section. We used the operational details of the collaborating companies as samples. The system was implemented with requirement (i) in training mode and requirements (ii) and (iii) in scoring mode.

In training mode, trainees wore an HMD and held the controller in both hands to perform operations in a virtual restaurant. In scoring mode, instructors could view replays of the trainees’ operations on a PC screen and provide comments.

The PoC system is implemented using the Unity game engine. For trainees, the resolution of the VR display was the same as the Quest 2 (1832 × 1920 pixels per eye). For instructors, the scoring mode was displayed in full HD on a PC screen.

Regarding the system configuration, while it is possible to construct a system that interacts in the VR space using a 2D monitor and mouse, this study focuses on training in “awareness” and “prioritization”. That is, tasks within the store must be managed while quickly moving one’s head to understand the situation in the restaurant. For this reason, the use of an HMD was considered appropriate.

### Training mode

**Fig. 6:**
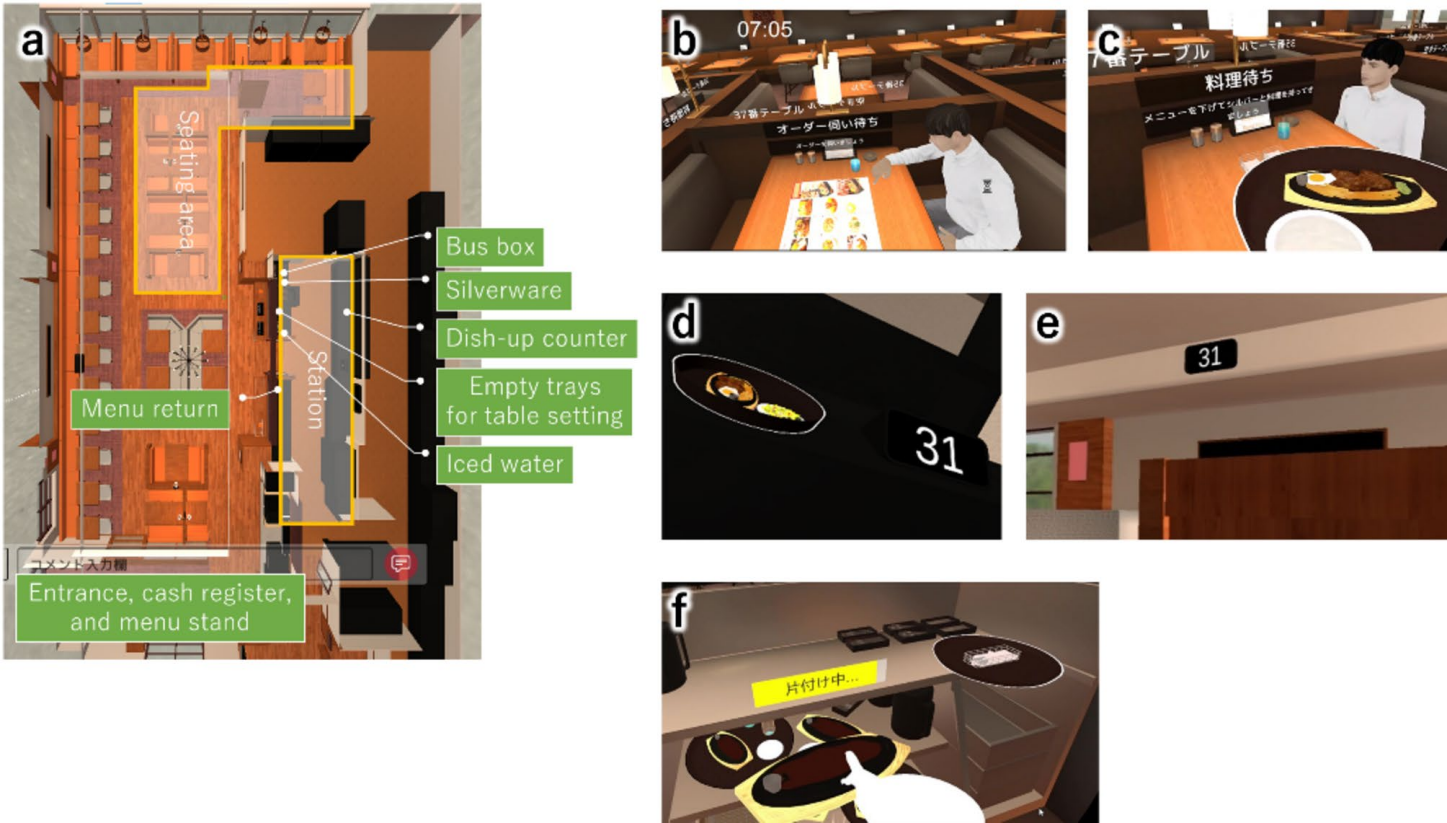
(**a**) Floor layout. Trainee performing tasks in VR restaurant. (**b**) Taking orders. (**c**) Serving meals. (**d** & **e**) Display of destination tables for dishes at (**d**) dish-up counter and (**e**) dining area. (**f**) Trainee cleans empty dishes.

The implemented training contents were located in the dining area, at the station (an employee-only area where cold water and silverware are available), and at the dish-up counter (an area where completed dishes are received from the kitchen). Figure 6 (a) shows the floor configuration. The VR restaurant was created from 3D scan data and drawings of the actual restaurant.

The operational procedures implemented are as shown.

1. Reception & hosting: Virtual customers wait at the entrance after arriving. Trainees meet customers and freely select an available seat each time to guide them (Refer to Fig. 6 (a) for the seating area). In actual restaurants, vibrations from devices worn by staff are used to notify them of the arrival of customers, thus we implemented this by making the controllers vibrate.
2. Providing menus and serving ice water: After the customers are seated, menus are brought out from a designated storage area and provided to customers, then ice water is carried out from the station and served.
3. Taking orders: To create the effect of customers deciding on their order, after a certain amount of time looking at the menu, customers take their eyes off the menu. The trainees then move to the tables to take orders (Fig. 6 (b)). The trainees then collect the menus and return them to the designated location. To simplify the training, only one type of meal is provided.
4. Meal preparation: Trainees bring silverware from the station to the table (forks, knives, etc.).
5. Serving meals: A certain amount of time after the order is placed, the meal is placed on the dish-up counter and served to the designated table (Fig. 6 (c)). The controllers give the vibration alert that the meal is ready, and the designated table is displayed at the dish-up counter and near the ceiling of the dining area (Fig. 6 (d)(e)).
6. Replacing ice water: The customers occasionally drink their ice water; when it is running low, trainees bring ice water from the station and replace the water glasses with new ones, putting away the old glasses in a designated place in the station.
7. Intermediate bussing: After the meal is served, customers finish eating within a certain amount of time, after which the trainees collect the utensils and store them in a designated location (bus box) in the station (Fig. 6 (f)).
8. Payment: After a certain amount of time has passed following the customers finishing the meal, the customers move to the cash register. The payment process is simulated by allowing the trainees to use the VR controller to point at an select the customers who approach the cash register area.
9. Table setting: After customers leave the restaurant, trainees bring empty trays from the station to clear their tables and to store the collected utensils in the designated location in the station.

In training mode, as mentioned above, trainees wore an HMD and held the controller in both hands. Regarding movement within the VR space, although teleportation is often used in VR, the goal of this study was to train awareness and priority-setting skills in a busy restaurant environment. To closely simulate the actual task of “walking around while keeping an eye on various parts of the restaurant”, it was necessary to move from table to table, and to move around the restaurant floor rather than using teleportation. The trainees could move forward, backward, left, and right in the virtual space, using the left stick, and could change the direction of their observation in the virtual space by naturally moving their heads or using the right stick to control the direction they were facing.

Pulling the trigger allowed trainees to hold the selected items such as the menu and dishes. Instructors observed the training in real-time on a PC screen. The head and hand positions of trainees and events that occurred in the restaurant were logged and saved to reproduce the training situations later on.

The models of each training event and vignette were consistent every time. However, the training was tailored to the skill level of the trainee by changing the level of difficulty, a parameter that modified the interval between customers entering the restaurant. Three levels of difficulty were established after consulting with partner companies: easy (simulating slow periods), normal, and hard (simulating peak hours), with the interval between customers entering the restaurant set to 180, 120, and 100 s, respectively.

**Fig. 7:**
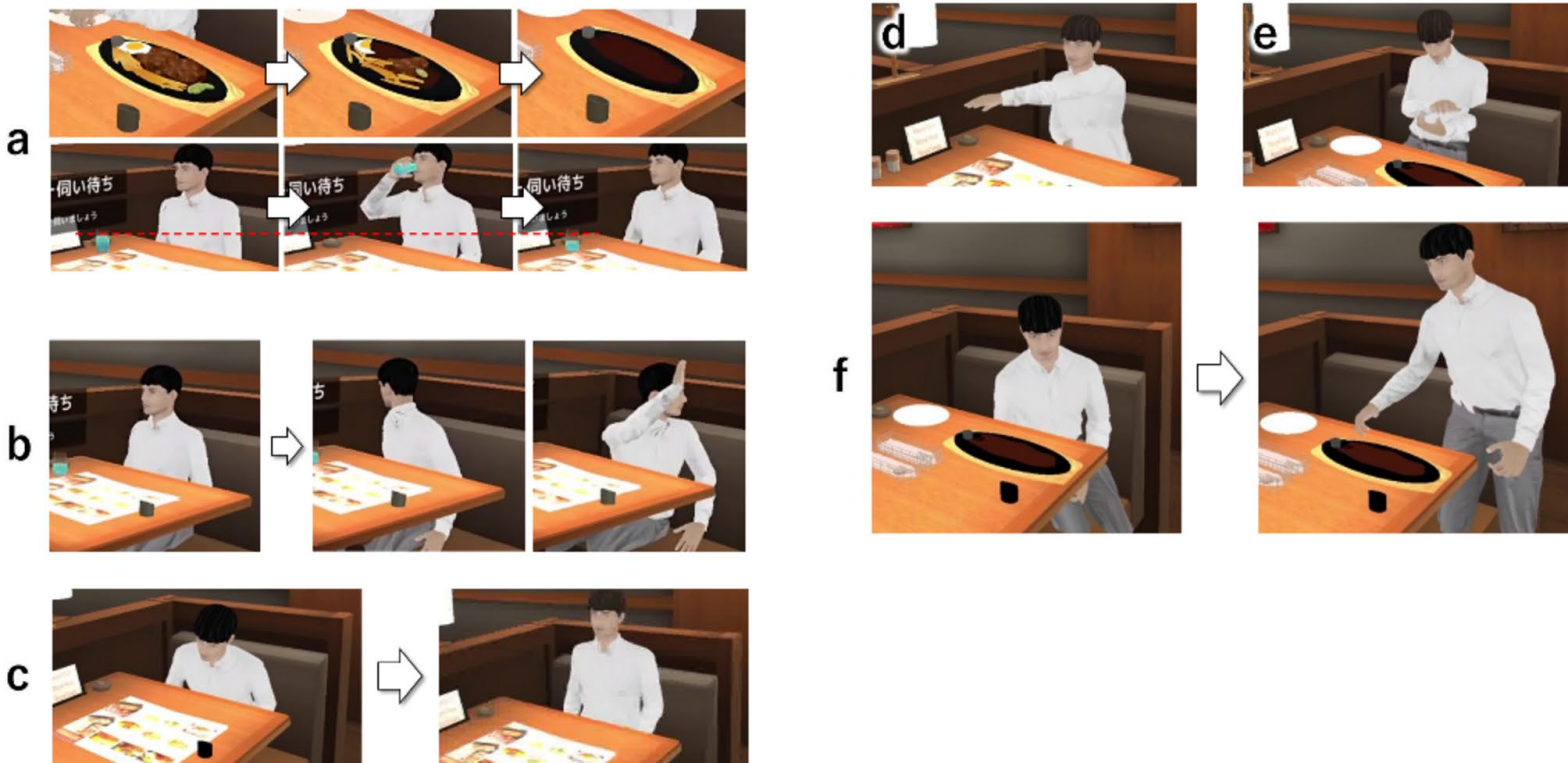
(**a**) Decrease in food and water over time. (**b**) Transition of customer waiting actions. Attention cue behaviors. (**c**) Customers looking up after deciding what to order. (**d**) After deciding their order, customers ring the bell. (**e**) Customers check their phone after eating. (**f**) Customers pick up the check when leaving.

To facilitate prioritization, the situation inside the VR restaurant was updated in a variety of ways. The progress of meals was displayed in three stages, and the ice water levels decreased each time the customers made drinking movements (Fig. 7 (a)). When the trainees delayed in responding and made customers wait for too long, the customers made movements to indicate that they were waiting, such as looking around and raising their hands (Fig. 7 (b)). The times at which customers exhibited waiting actions due to frustration, the time it took for customers to decide on their orders, the time the customer waited until the meal was ready, and the time it took for customers to finish their meals were all determined in consultation with the head of training at the partner restaurant company.

Furthermore, recordings of customer behavior at actual restaurants^23^ show that customers look up (Fig. 7 (c)) and ring the bell when they decide to order (Fig. 7 (d)), look at their phones (Fig. 7 (e)) after finishing their meal, and grab the bill when they get up from their seats (Fig. 7 (f)), all of which were implemented in the system.

Regarding the events during training, our training system focuses on mastering fundamental operations and excluded unusual responses (such as customers dropping silverware or spilling water) from its scope. This is similar to driving a car—once trainees have mastered the basics, they could allocate more attention to handling unusual situations.

#### Scoring mode

Customer satisfaction was calculated based on the quantitative evaluation metrics:

1. Reception & Hosting: From the time the customers enter the restaurant to the time they are shown their table by the trainee.
2. Menus provided & Ice water served: From time customers are seated to time they are served the ice water.
3. Taking orders: From the time customers decide their order to the time trainees go to the table and take the order.
4. Meal preparation: No points added or deducted due to time considerations.
5. Serving meals: From the time food is ready at the dish-up counter to the time it is served.
6. Replacing ice water glasses: From the time the glass becomes empty to the time it is replaced with a new one.
7. Intermediate bussing: No points added or deducted due to time considerations.
8. Payment: From the time the customer leaves their seat to the time payment is handled.
9. Table setting: No points added or deducted due to time considerations.

Specifically, for items (1) to (3), (5), (6), and (8), points were deducted if the predetermined time was exceeded and the customers were made to wait. There were two levels of point deduction. No points were added or subtracted for time for meal preparation, (4), or table setting, (9). However, for (4), points were deducted for forgetting to perform the next step of serving meals. The system was configured to prevent trainees from hosting the next customers until they had performed (9). Additionally, customer satisfaction points were added when trainees provided customers with above-average service. Here, points were added when: ice water glasses were replaced with new ones when the water level was between half-full and empty for (6), intermediate bussing was completed by the time the customers left the table for (7), and when the trainee decided to go to the cash register in advance of the customer and prepare the bill before the customer arrived for (8). The points were added and subtracted in one-point increments.

**Fig. 8:**
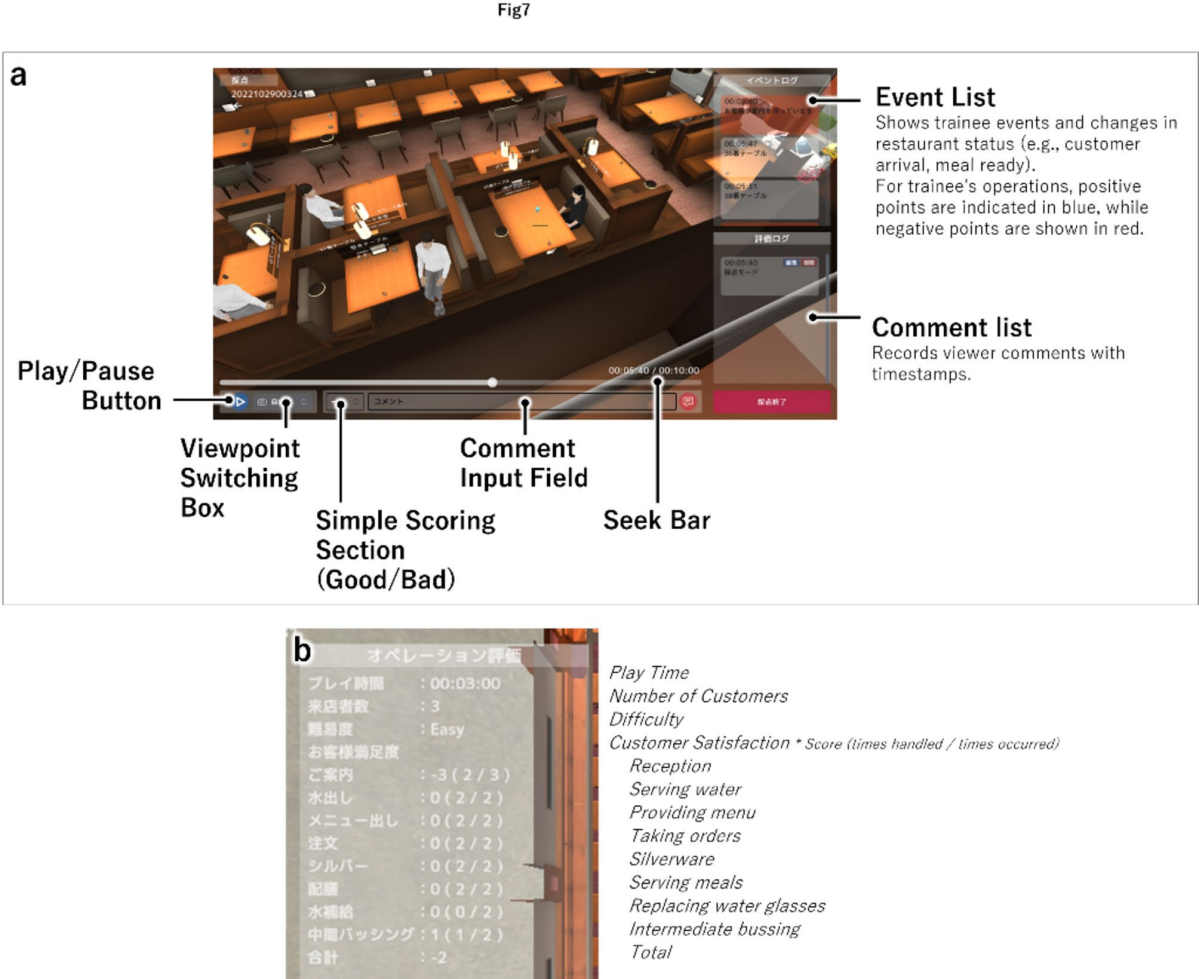
(**a**) Scoring screen (replay). (**b**) Example of training summary.

Figure 8 (a) shows an example of the implemented system in scoring mode. It is displayed in full screen on the PC. The instructor can check the training progress by manipulating the viewpoint (at the bottom of Fig. 1, an example of viewpoint switching is shown) and timeline and can add comments at any time. Additionally, a summary was implemented to display the number of occurrences and the number of points deducted (or added) for each item (Fig. 8 (b)). Furthermore, this summary was also shown to the trainee at the end of the training session in training mode.

## Experiment

### Experimental design and procedure

#### Experts

After they practiced operating the system, the experts engaged in training mode for 10 minutes and then reflected on their experience using the scoring mode. Then, they scored the sample training data that had been prepared in advance. The experts were instructed to review the training data from the perspective of “If I were this person’s instructor, what guidance would I provide?” and provide comments to the trainee accordingly. A maximum of 20 minutes was allotted for reflection and scoring, and participants reported when they finished.

#### Novices

After they practiced operating the system, they repeated the 10-minute training sessions and reflections for five days. Note that because the experiment was conducted in accordance with each participant’s schedule, it was not necessarily conducted over five consecutive days. All participants began at the “easy”, level of difficulty, and when participants had completed each level of difficulty with a total score above zero, the difficulty for the next training session was raised to the next level.

**Fig. 9:**
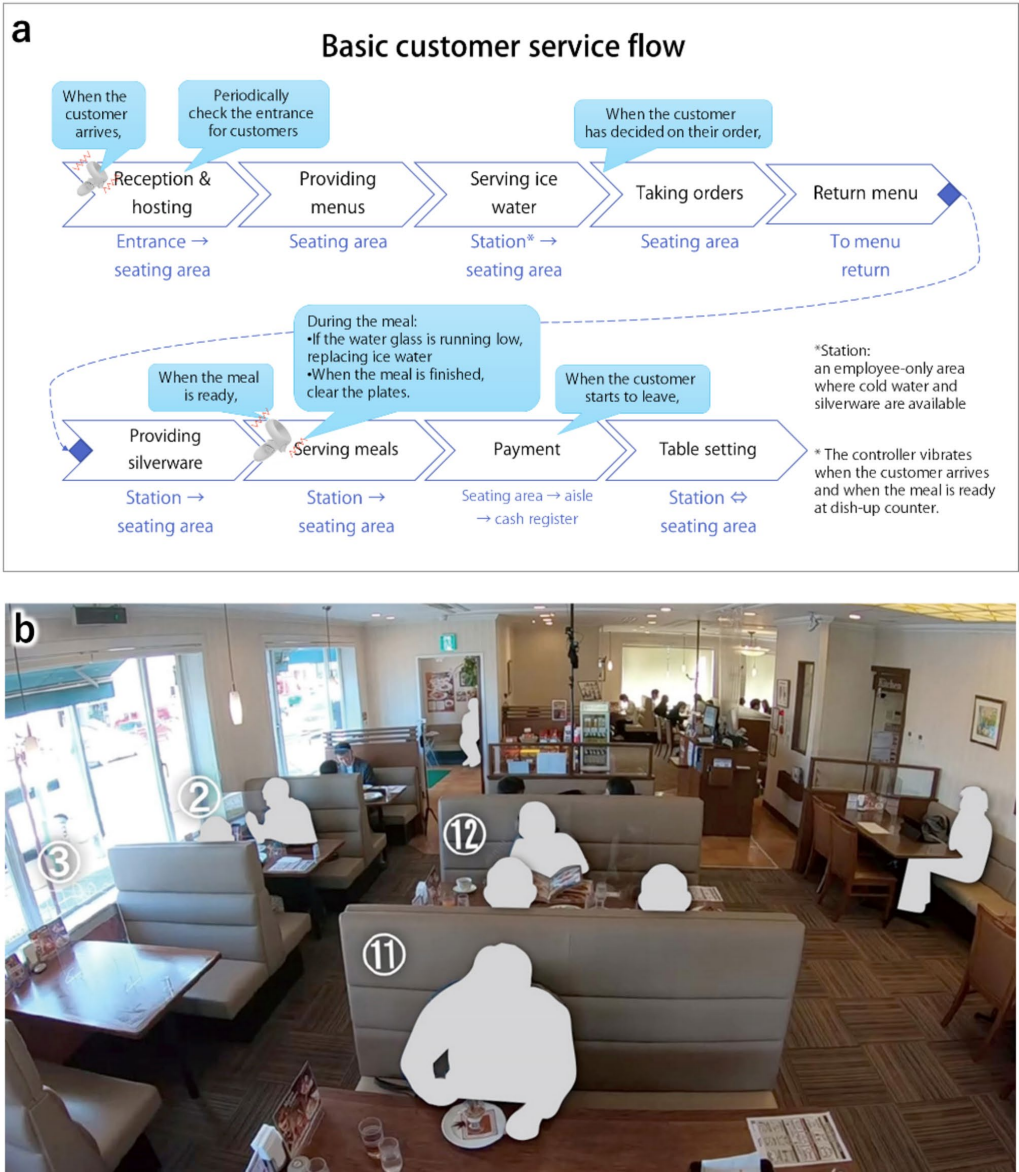
(**a**) Diagram explaining basic customer service flow as presented to participants. (**b**) Reconstructed illustration of the example video used in the awareness test, with human figures replaced by silhouettes to protect personal identity.

Before training on the first day, the customer service flow for one customer was illustrated (Fig. 9 (a)) and participants read it thoroughly until they understood it. The trainee followed this flow, just as in real-world operations, and performed customer service tasks in parallel for multiple customers who arrived at different times. As mentioned, the models of each training event and vignette were consistent every time. This diagram was also presented to participants upon request from the second day onwards.

Next, participants watched a 5-minute video shot at an actual restaurant^23^ and conducted an “awareness test” to measure the number of “points to be aware of at work” that they noticed in the video. Participants were instructed as follows: “When providing service to customers in a restaurant, if you notice customer behavior or a situation that you think is important to customer service, pause the video for a moment, then tell us what you would do next while pointing it out with the mouse pointer (e.g., “Customers came to the entrance so I proceeded to greet them.“). Figure 9 (b) shows the example video used in the awareness test. Participants were informed in advance of the locations of the entrance and cash register in the video, and the seat numbers were displayed in the video in text. To better simulate the work conditions, rewinding and fast-forwarding were not allowed and the progress of participants in answering as well as the on-screen situation was recorded. An awareness test was also conducted after the experience on the final day, and videos from different cameras were used on the first and final days. Participants were not allowed to check their answers in either test. Multiple videos were prepared for the awareness test and randomly assigned to each participant. Under the supervision of the head of education of the partner restaurant company, between 9 and 14 points to be aware of were selected for each video.

This study was approved by the Committee for Ergonomic Experiments at AIST (approval number: 2020–1019 F). All methods were performed in accordance with relevant guidelines and regulations, including the Declaration of Helsinki. Written informed consent, including consent to publish identifying information and images, was obtained from all participants.

### Experimental equipment

A Mouse Computer G-tune E5-165 PC (CPU: Intel’s Core i7-11800 H 2.3 GHz; memory: 32GB; GPU: Windows 10 PC equipped with Nvidia’s GeForce RTX 3060/6GB GDDR6) was used for the experiment.

### Measurement items

The details of the measurements conducted in this experiment are shown in Table 1. The evaluation metrics for novices included feelings of resistance to customer service, system usability scale (SUS)^17^, and awareness tests were conducted on the first and final day (Day 5) to measure how participants changed before and after training. In addition, NASA-TLX (Task load index)^21^ was measured on Days 1, 3, and 5 to measure changes in load during training. The level of difficulty was recorded each time, whereas the rest of the items were measured on Day 5.

To measure the number of things participants noticed in their training mode experiences, they were asked after each experience to list in the free description as many “points important to customer service” they had noticed during the experience and as they could think of. In calculating the totals, the following seven points in accordance with the customer service flow (see Sect. “Training mode”) were counted as correct answers:

1. Entering the restaurant: customers are standing at the entrance, looking around, raising their hands, vibration alert.
2. Order: customers look up from the menu, press the bell, look around, raise a hand.
3. Serving Meals: Meal is out at the dish-up counter, vibration alert, customers fidget, looking around, raise hands.
4. Replacing ice water glasses: ice water levels are low.
5. Intermediate bussing (clear some tableware): plates are empty.
6. Leaving and payment: customers leave their seats, walk down the aisle, the customer is at the cash register.
7. Table setting: customers leave, plates are empty.

Participants’ free descriptions were analyzed, and the number of responses that could be interpreted as including correct answers was then totaled.

**Table 1:** Evaluation metrics.

	Items	Expert		Novice
Subjective evaluation	Usefulness of training mode (5-level evaluation, free description)	✔		✔
	Usefulness of self-reflection in scoring mode (5-level evaluation, free description)	✔		✔
	Usefulness of scoring others in scoring mode (5-level evaluation, free description)	✔		✔
	Feelings of resistance towards customer service (5-level evaluation, free description)			✔
	System Usability Scale (SUS) [17]	✔		✔
	iGroup Presence Questionnaire (IPQ) [19]			✔
	NASA-TLX (Task load index) [21]			✔
Objective evaluation	Awareness test: Finding rate			✔
	Awareness test: Time to find			✔
	Number of points trainee became aware of during training mode experience			✔
	Changes in difficulty level			✔
		Expert		Novice
		Self reflection	Scoring sample data	Self reflection
Objective evaluation	Scoring mode: No. of Comments & Comment Type	✔	✔	✔
	Scoring mode: Scoring time	✔	✔	✔
	Scoring mode: Number and duration of viewpoint changes	✔	✔	✔

## Data Availability

The data are not publicly available due to their containing information that could compromise the privacy of research participants.
